# Activation of Dopamine Receptors in the Nucleus Accumbens Promotes Sucrose-Reinforced Cued Approach Behavior

**DOI:** 10.3389/fnbeh.2016.00144

**Published:** 2016-07-14

**Authors:** Johann du Hoffmann, Saleem M. Nicola

**Affiliations:** Department of Neuroscience and Psychiatry, Albert Einstein College of MedicineBronx, NY, USA

**Keywords:** reward-seeking behavior, mesolimbic, locomotion, satiety, obesity, addiction, extinction

## Abstract

Dopamine receptor activation in the nucleus accumbens (NAc) promotes vigorous environmentally-cued food-seeking in hungry rats. Rats fed *ad libitum*, however, respond to fewer food-predictive cues, particularly when the value of food reward is low. Here, we investigated whether this difference could be due to differences in the degree of dopamine receptor activation in the NAc. First, we observed that although rats given *ad libitum* access to chow in their home cages approached a food receptacle in response to reward-predictive cues, the number of such approaches declined as animals accumulated food rewards. Intriguingly, cued approach to food occurred in clusters, with several cued responses followed by successive non-responses. This pattern suggested that behavior was dictated by transitions between two states, responsive and non-responsive. Injection of D1 or D2 dopamine receptor agonists into the NAc dose-dependently increased cue responding by promoting transitions to the responsive state and by preventing transitions to the non-responsive state. In contrast, antagonists of either D1 or D2 receptors promoted long bouts of non-responding by inducing transitions to the non-responsive state and by preventing transitions to the responsive state. Moreover, locomotor behavior during the inter-trial interval was correlated with the responsive state, and was also increased by dopamine receptor agonists. These results suggest that activation of NAc dopamine receptors plays an important role in regulating the probability of approach to food under conditions of normative satiety.

## Introduction

For a hungry animal, the decision to respond to a food-predictive cue is a trivial one. Hungry, well-trained animals respond to nearly every cue signaling food availability. The likelihood and vigor of these responses, however, is lower in the normative state of satiety. What are the neural mechanisms that set the probability of approach to food under such conditions? Because responding to food-predictive cues in the absence of caloric need likely contributes to elevated calorie consumption (Boulos et al., 2012; Boyland and Halford, 2013), answering this question is an important step toward understanding both normal caloric intake and dysregulated intake in obesity.

We began with the observation that dopamine receptor activation in the nucleus accumbens (NAc) is critical for cued approach toward food-associated objects under conditions where a rat's starting position varies from trial to trial. Under these conditions, injection of either D1 or D2 dopamine receptor antagonists into the NAc core reduces the proportion of cues to which animals respond by increasing the latency to initiate approach (Nicola, 2010). These effects result from a reduction in the magnitude and prevalence of dopamine-dependent cue-evoked excitations (du Hoffmann and Nicola, 2014). These excitations, which are observed in nearly half of NAc neurons, precede movement onset and are greater when the latency to initiate movement is shorter (McGinty et al., 2013; du Hoffmann and Nicola, 2014; Morrison and Nicola, 2014). One hypothesis to explain reduced cue responding in non-food-restricted animals is that less dopamine is released in less hungry animals, an idea supported by electrochemical, microdialysis and electrophysiological evidence (Ostlund et al., 2011; Branch et al., 2013; Cone et al., 2014). Consequently, there may be less activation of NAc dopamine receptors under conditions of relative satiety, leading to a lower probability of responding to food-associated cues.

To test this hypothesis, we asked whether pharmacologically blocking and tonically activating NAc dopamine receptors in non-food-restricted animals could, respectively, attenuate and promote cue responding. In the experimental phase, rats had access to food and water *ad libitum* in their home cages in order to induce a state of relative satiety, which greatly decreased the probability that animals would respond to a given cue presentation. This lower response probability allowed us to assess whether dopamine receptor agonists increase that probability, which is not possible in hungry animals because they respond to nearly every cue. We found that blocking dopamine receptors decreased responding whereas activation of the same receptors increased responding. These results suggest that response probability and food seeking in relatively sated animals is actively regulated by NAc dopamine.

## Materials and methods

### Animals

Eight male Long-Evans that weighed 275–300 g were purchased from Harlan and singly housed on a 12 h light/ dark cycle. All experiments were conducted in the light phase. Animal care was identical to previously published accounts (Nicola, 2010; du Hoffmann et al., 2011; McGinty et al., 2013; du Hoffmann and Nicola, 2014; Morrison and Nicola, 2014). Upon arrival, rats were given 1 week of rest and were then habituated to being handled by the experimenter. After habituation, animals were food restriced to ~90% of free feeding body weight prior to beginning the initial stages of training. After the early stages of training, animals were given free access to standard lab chow in their home cage. All animal procedures were consistent with the U.S National Institutes of Health *Guide for the Care and Use of Laboratory Animals* and were approved by the Institutional Animal Care and Use Committee at Albert Einstein College of Medicine.

### Operant chambers

Behavioral training took place in operant chambers (30 × 25 cm) purchased from Med Associates. Experiments were conducted in sound-attenuating cabinets with blue house lights illuminated. A constant white noise (65 dB) was played within the chamber to limit distractions from outside noise. Operant chambers were equipped with a reward receptacle on one wall. A photobeam located across the front of the receptacle measured receptacle entry and exit times. A syringe pump, located outside the chamber, was used to deliver liquid sucrose reward into the reward receptacle. Behavioral time stamps were recorded with a resolution of 1 ms.

### 2CS task training

Animals were food restricted during the initial training stages. The first stage of training required that the animals enter the food receptacle, which triggered delivery of 10% liquid sucrose. After a 10 s delay to allow for reward consumption, animals had to leave the receptacle and re-enter it in order to earn additional reward. In subsequent training stages, delays of 20 s and then 30 s were introduced between reward availability. Criterion performance was set at 100 rewards earned in 1 h. After criterion performance was established with a 30 s delay between reward availability, two auditory cues were introduced that predicted either a small or large reward (150 or 250 μl of 10% sucrose solution in water). The auditory cues consisted of a siren tone (which cycled in frequency from 4 to 8 kHz over 400 ms) and an intermittent tone (6 kHz tone on for 40 ms, off for 50 ms); cues were assigned to large and small reward randomly for each rat and the cue-reward magnitude relationship remained constant across training and experiments for a given rat. Reward delivery was contingent on the rat entering the reward receptacle during the cue presentation, at which point the cue was terminated. Cues were on for up to 5 s. The inter-trial interval was chosen pseudorandomly from a truncated exponential distribution with a mean of 30 s. Once animals responded to > 80% of the cues, animals were fed *ad libitum* in their home cages from that point until the end of experiments. After task performance stabilized, the sucrose concentration of the liquid reward was reduced from 10% to 3%; the volumes were not changed. Behavior was monitored daily until asymptotic task performance was achieved.

### Surgery

After behavioral performance stabilized, bilateral guide cannulae targeting the NAc core were chronically implanted as described previously (Nicola, 2010; Lardeux et al., 2015). Briefly, animals were anesthetized with isofluorane and placed into a stereotaxic frame with the head flat. Small holes were drilled bilaterally in the skull at 1.4 mm anterior and ± 1.5 mm lateral from Bregma. A stereotaxic arm was used to precisely place the cannulae into these holes and then lower them into the brain to a final depth of 6 mm from the top of the skull (2 mm above the NAc). Cannulae were held in place with bone screws and dental cement. Two threaded posts were placed vertically on the skull and embedded in dental cement. These posts interfaced with screws to a head stage containing two LEDs, which allowed automated video tracking during experiments. Animals received the antibiotic enrofloxacin prior to and 1 day post-surgery. After surgery, rats were given 1 week to recover before a brief post-surgical retraining period on the 2CS task began.

### Drugs

Drugs were purchased from Sigma and freshly dissolved in 0.9% sterile saline on the day they were used. Drug doses per side were: “D1 agonist low,” 0.1 μg SKF81297; “D1 agonist high,” 0.4 μg SKF81297; “D1 antagonist,” 1.1 μg Schering 23390; “D2 agonist low,” 1 μg quinpirole; “D2 agonist high,” 10 μg quinpirole; “D2 antagonist,” 2.2 μg raclopride.

### Microinjection procedure

As previously described (Nicola, 2010; Lardeux et al., 2015), rats were gently restrained with a towel while 33 ga injectors were inserted into the guide cannulae such that the injector extended 2 mm further ventral from the bottom of the guide, reaching the center of the NAc core. After 1 min, 0.5 μL of drug solution was injected over 2 min with a precision syringe pump. Drugs were given 1 min to diffuse, after which the animals were immediately placed into the operant chambers. The order of drug injections was randomized across rats. Injections were performed twice per week (on Tuesdays and either Thursdays or Fridays), with an intervening uninjected session run on the day prior to each injection to ensure that behavior recovered from the previous injection.

### Video tracking

On test days, the rat's position was recorded using an overhead camera (30 frames/s) and automated tracking system (either Plexon Cineplex or Noldus Ethovision). This system tracked the x and y positions of red and green LEDs attached to the rat's head. As previously described (Nicola, 2010; McGinty et al., 2013; du Hoffmann and Nicola, 2014; Morrison and Nicola, 2014), to determine the rat's position in the operant chamber we calculated a centroid (the center point) between the LEDs for each video frame. Missing positions up to 10 successive frames were linearly interpolated; if > 10 successive frames were missing the data were discarded. For each frame, we then calculated the SD of the distances of centroid positions within a temporal window of 200 ms. When log transformed, these SD values were bimodally distributed, with the lower peak representing epochs of non-movement and the upper peak movement. We then fit two Gaussian functions to these distributions and the movement threshold was determined as the point where the upper and lower distributions overlapped the least. Movement was defined as 8 consecutive frames above this threshold.

### Data analysis

One rat failed to re-attain pre-surgery performance levels after cannula implantation and thus was not subjected to microinjections. The cannulae from a second rat became clogged and consequently some microinjections were not performed. Thus, data were obtained from 7 microinjections for some experiments and 6 for others. Behavioral time stamps and raw video tracking position data were exported and analysis was performed with custom routines in the R statistical computing environment (R Core Team, 2013).

**Figure 1:**
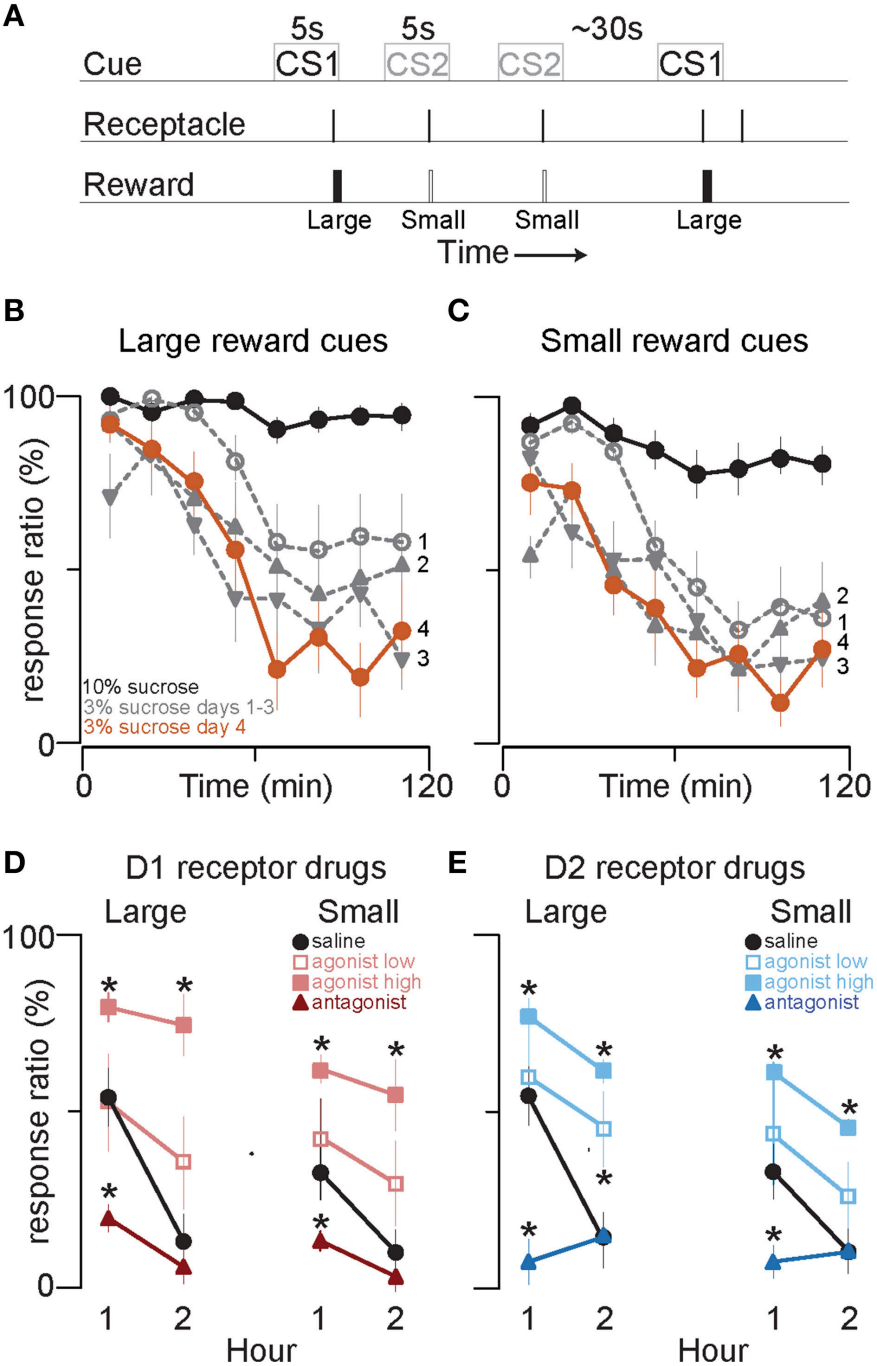
**D1 and D2 receptor agonists and antagonists, respectively promote and attenuate cued approach to reward**. **(A)** 2CS+ task schematic. Time is not to scale. **(B,C)** Single session average response ratio (% of cues responded to) in 15 min time bins to cues that predict large **(B)** and small **(C)** volumes of 10% sucrose (black line), and the first three (gray) and fourth (orange) days after the switch to equivalent volumes of 3% sucrose reward. **(D)** Symbols show the mean response ratios to cues that predict 250 μl (“Large”) or 150 μl (“Small”) of 3% sucrose reward after injection of saline (black circles), low (red open squares) and high doses (light red squares) of the D1 receptor agonist SKF 81927, and of the D1 antagonist SCH 23390 (dark red triangles), into the NAc core. The bold lines connect the response ratio data from the first and second hour of the behavioral session. **(E)** The graph follows identical conventions to those in **(D)** for injection of saline (black circles), low (blue open squares) and high dose D2 receptor agonist quinpirole (light blue squares) and D2 antagonist raclopride (dark blue triangles) into the NAc core. Error bars in this and all subsequent figures denote SEM. ^*^p < 0.05 compared to saline. See Table 1 for all statistical results. See Methods Section Drugs for drug doses.

In Figures 1B–E, we calculated the cue response ratio by dividing the number of cues responded to by the number of cues presented in 15 min or 1 h bins and plotted them as cross-session means. To assess task variables that influence performance in each drug, we used repeated measures ANOVA with response ratio as the dependent variable against two factors, time interval (1 and 2 h) and cue type (large and small). *Post-hoc* two-tailed paired *t*-tests were used within each drug condition to test whether session time and cue type (large and small) significantly influenced response ratio. Two-tailed Welch's *t*-tests were used to compare response ratios for each drug to saline. P values for *post-hoc t*-tests were corrected using the Sidak multiple comparisons correction procedure. The significance threshold for all statistical tests was set at *p* < 0.05. The results from all statistical tests can be found in Table 1.

**Table 1:** **Statistical results**.

**Figure**	**Drug (s)**	**Variables**	**Event**	**Test**	**Result**
Figures 1D,E	Saline	hour	Response (%)	Repeated measures ANOVA	*F*_(1, 6)_ = 46.02, *P* ≤ 0.001
Figures 1D,E	Saline	cue type	Response (%)	Repeated measures ANOVA	*F*_(1, 6)_ = 46.72, *P* ≤ 0.001
Figures 1D,E	Saline	hour × cue type	Response (%)	Repeated measures ANOVA	*F*_(1, 6)_ = 19.87, *P* = 0.004
Figures 1D,E	Saline	Large 1 h vs. Large 2 h	Response (%)	paired *t*-test	*t*_(6)_ = 6.339, *P* ≤ 0.001
Figures 1D,E	Saline	Small 1 h vs. Small 2 h	Response (%)	paired *t*-test	*t*_(6)_ = 6.928, *P* ≤ 0.001
Figures 1D,E	Saline	Large 1 h vs. Small 1 h	Response (%)	paired *t*-test	*t*_(6)_ = 6.312, *P* ≤ 0.001
Figures 1D,E	Saline	Large 2 h vs. Small 2 h	Response (%)	paired *t*-test	*t*_(6)_ = 1.334, *P* = 0.231
Figure 1D	D1 agonist low dose	hour	Response (%)	Repeated measures ANOVA	*F*_(1, 6)_ = 3.28, *P* = 0.12
Figure 1D	D1 agonist low dose	cue type	Response (%)	Repeated measures ANOVA	*F*_(1, 6)_ = 5.81, *P* = 0.053
Figure 1D	D1 agonist low dose	hour × cue type	Response (%)	Repeated measures ANOVA	*F*_(1, 6)_ = 0.66, *P* = 0.448
Figure 1D	D1 agonist high dose	hour	Response (%)	Repeated measures ANOVA	*F*_(1, 5)_ = 1.98, *P* = 0.219
Figure 1D	D1 agonist high dose	cue type	Response (%)	Repeated measures ANOVA	*F*_(1, 5)_ = 28.27, *P* = 0.003
Figure 1D	D1 agonist high dose	hour × cue type	Response (%)	Repeated measures ANOVA	*F*_(1, 5)_ = 0, *P* = 0.978
Figure 1D	D1 antagonist	hour	Response (%)	Repeated measures ANOVA	*F*_(1, 6)_ = 3.88, *P* = 0.096
Figure 1D	D1 antagonist	cue type	Response (%)	Repeated measures ANOVA	*F*_(1, 6)_ = 5.01, *P* = 0.066
Figure 1D	D1 agonist low dose	Large 1 h vs. Large 2 h	Response (%)	paired *t*-test	*t*_(6)_ = 1.632, *P* = 0.154
Figure 1D	D1 agonist low dose	Small 1 h vs. Small 2 h	Response (%)	paired *t*-test	*t*_(6)_ = 2.031, *P* = 0.089
Figure 1D	D1 agonist low dose	Large 1 h vs. Small 1 h	Response (%)	paired *t*-test	*t*_(6)_ = 2.106, *P* = 0.08
Figure 1D	D1 agonist low dose	Large 2 h vs. Small 2 h	Response (%)	paired *t*-test	*t*_(6)_ = 1.732, *P* = 0.134
Figure 1D	Saline vs. D1 agonist low dose	Large 1 h	Response (%)	Sidak corrected Welch's *t*-test	*t*_(12)_ = 0.231, *P* = 1
Figure 1D	Saline vs. D1 agonist low dose	Small 1 h	Response (%)	Sidak corrected Welch's *t*-test	*t*_(12)_ = –0.841, *P* = 0.834
Figure 1D	Saline vs. D1 agonist low dose	Large 2 h	Response (%)	Sidak corrected Welch's *t*-test	*t*_(12)_ = –1.776, *P* = 0.303
Figure 1D	Saline vs. D1 agonist low dose	Small 2 h	Response (%)	Sidak corrected Welch's *t*-test	*t*_(12)_ = –1.706, *P* = 0.312
Figure 1D	D1 agonist high dose	Large 1 h vs. Large 2 h	Response (%)	paired *t*-test	*t*_(6)_ = 1.902, *P* = 0.106
Figure 1D	D1 agonist high dose	Small 1 h vs. Small 2 h	Response (%)	paired *t*-test	*t*_(6)_ = 0.844, *P* = 0.431
Figure 1D	D1 agonist high dose	Large 1 h vs. Small 1 h	Response (%)	paired *t*-test	*t*_(6)_ = 5.29, *P* = 0.002
Figure 1D	D1 agonist high dose	Large 2 h vs. Small 2 h	Response (%)	paired *t*-test	*t*_(6)_ = 3.179, *P* = 0.019
Figure 1D	Saline vs. D1 agonist high dose	Large 1 h	Response (%)	Sidak corrected Welch's *t*-test	*t*_(12)_ = –3.175, *P* = 0.024
Figure 1D	Saline vs. D1 agonist high dose	Small 1 h	Response (%)	Sidak corrected Welch's *t*-test	*t*_(12)_ = –3.54, *P* = 0.012
Figure 1D	Saline vs. D1 agonist high dose	Large 2 h	Response (%)	Sidak corrected Welch's *t*-test	*t*_(12)_ = –7.444, *P* ≤ 0.001
Figure 1D	Saline vs. D1 agonist high dose	Small 2 h	Response (%)	Sidak corrected Welch's *t*-test	*t*_(12)_ = –6.76, *P* ≤ 0.001
Figure 1D	D1 antagonist	Large 1 h vs. Large 2 h	Response (%)	paired *t*-test	*t*_(6)_ = 1.991, *P* = 0.094
Figure 1D	D1 antagonist	Small 1 h vs. Small 2 h	Response (%)	paired *t*-test	*t*_(6)_ = 1.825, *P* = 0.118
Figure 1D	D1 antagonist	Large 1 h vs. Small 1 h	Response (%)	paired *t*-test	*t*_(6)_ = 1.823, *P* = 0.118
Figure 1D	D1 antagonist	Large 2 h vs. Small 2 h	Response (%)	paired *t*-test	*t*_(6)_ = 2.749, *P* = 0.033
Figure 1D	Saline vs. D1 antagonist	Large 1 h	Response (%)	Sidak corrected Welch's *t*-test	*t*_(12)_ = 4.523, *P* = 0.005
Figure 1D	Saline vs. D1 antagonist	Small 1 h	Response (%)	Sidak corrected Welch's *t*-test	*t*_(12)_ = 3.791, *P* = 0.012
Figure 1D	Saline vs. D1 antagonist	Large 2 h	Response (%)	Sidak corrected Welch's *t*-test	*t*_(12)_ = 1.41, *P* = 0.368
Figure 1D	Saline vs. D1 antagonist	Small 2 h	Response (%)	Sidak corrected Welch's *t*-test	*t*_(12)_ = 2.063, *P* = 0.248
Figure 1E	D1 antagonist	hour × cue type	Response (%)	Repeated measures ANOVA	*F*_(1, 6)_ = 1.35, *P* = 0.289
Figure 1E	D2 agonist low dose	hour	Response (%)	Repeated measures ANOVA	*F*_(1, 5)_ = 6.65, *P* = 0.05
Figure 1E	D2 agonist low dose	cue type	Response (%)	Repeated measures ANOVA	*F*_(1, 5)_ = 15.34, *P* = 0.011
Figure 1E	D2 agonist low dose	hour × cue type	Response (%)	Repeated measures ANOVA	*F*_(1, 5)_ = 0.25, *P* = 0.64
Figure 1E	D2 agonist high dose	hour	Response (%)	Repeated measures ANOVA	*F*_(1, 5)_ = 2.44, *P* = 0.179
Figure 1E	D2 agonist high dose	cue type	Response (%)	Repeated measures ANOVA	*F*_(1, 5)_ = 43.31, *P* ≤ 0.001
Figure 1E	D2 agonist high dose	hour × cue type	Response (%)	Repeated measures ANOVA	*F*_(1, 5)_ = 0, *P* = 0.991
Figure 1E	D2 antagonist	hour	Response (%)	Repeated measures ANOVA	*F*_(1, 6)_ = 1.5, *P* = 0.266
Figure 1E	D2 antagonist	cue type	Response (%)	Repeated measures ANOVA	*F*_(1, 6)_ = 0.13, *P* = 0.735
Figure 1E	D2 antagonist	hour × cue type	Response (%)	Repeated measures ANOVA	*F*_(1, 6)_ = 2.44, *P* = 0.169
Figure 1E	D2 agonist low dose	Large 1 h vs. Large 2 h	Response (%)	paired *t*-test	*t*_(5)_ = 1.988, *P* = 0.103
Figure 1E	D2 agonist low dose	Small 1 h vs. Small 2 h	Response (%)	paired *t*-test	*t*_(5)_ = 2.641, *P* = 0.046
Figure 1E	D2 agonist low dose	Large 1 h vs. Small 1 h	Response (%)	paired *t*-test	*t*_(5)_ = 3.224, *P* = 0.023
Figure 1E	D2 agonist low dose	Large 2 h vs. Small 2 h	Response (%)	paired *t*-test	*t*_(5)_ = 3.173, *P* = 0.025
Figure 1E	Saline vs. D2 agonist low dose	Large 1 h	Response (%)	Sidak corrected Welch's *t*-test	*t*_(11)_ = –0.283, *P* = 1
Figure 1E	Saline vs. D2 agonist low dose	Small 1 h	Response (%)	Sidak corrected Welch's *t*-test	*t*_(11)_ = –0.764, *P* = 0.834
Figure 1E	Saline vs. D2 agonist low dose	Large 2 h	Response (%)	Sidak corrected Welch's *t*-test	*t*_(11)_ = –3.164, *P* = 0.036
Figure 1E	Saline vs. D2 agonist low dose	Small 2 h	Response (%)	Sidak corrected Welch's *t*-test	*t*_(11)_ = –1.773, *P* = 0.312
Figure 1E	D2 agonist high dose	Large 1 h vs. Large 2 h	Response (%)	paired *t*-test	*t*_(5)_ = 1.652, *P* = 0.159
Figure 1E	D2 agonist high dose	Small 1 h vs. Small 2 h	Response (%)	paired *t*-test	*t*_(5)_ = 1.289, *P* = 0.254
Figure 1E	D2 agonist high dose	Large 1 h vs. Small 1 h	Response (%)	paired *t*-test	*t*_(5)_ = 3.552, *P* = 0.016
Figure 1E	D2 agonist high dose	Large 2 h vs. Small 2 h	Response (%)	paired *t*-test	*t*_(5)_ = 3.012, *P* = 0.03
Figure 1E	Saline vs. D2 agonist high dose	Large 1 h	Response (%)	Sidak corrected Welch's *t*-test	*t*_(11)_ = –3.766, *P* = 0.012
Figure 1E	Saline vs. D2 agonist high dose	Small 1 h	Response (%)	Sidak corrected Welch's *t*-test	*t*_(11)_ = –6.149, *P* ≤ 0.001
Figure 1E	Saline vs. D2 agonist high dose	Large 2 h	Response (%)	Sidak corrected Welch's *t*-test	*t*_(11)_ = –5.706, *P* ≤ 0.001
Figure 1E	Saline vs. D2 agonist high dose	Small 2 h	Response (%)	Sidak corrected Welch's *t*-test	*t*_(11)_ = –3.797, *P* = 0.015
Figure 1E	D2 antagonist	Large 1 h vs. Large 2 h	Response (%)	paired *t*-test	*t*_(6)_ = –1.608, *P* = 0.159
Figure 1E	D2 antagonist	Small 1 h vs. Small 2 h	Response (%)	paired *t*-test	*t*_(6)_ = –0.76, *P* = 0.476
Figure 1E	D2 antagonist	Large 1 h vs. Small 1 h	Response (%)	paired *t*-test	*t*_(6)_ = –0.718, *P* = 0.5
Figure 1E	D2 antagonist	Large 2 h vs. Small 2 h	Response (%)	paired *t*-test	*t*_(6)_ = 1.472, *P* = 0.191
Figure 1E	Saline vs. D2 antagonist	Large 1 h	Response (%)	Sidak corrected Welch's *t*-test	*t*_(12)_ = 9.966, *P* ≤ 0.001
Figure 1E	Saline vs. D2 antagonist	Small 1 h	Response (%)	Sidak corrected Welch's *t*-test	*t*_(12)_ = 6.352, *P* ≤ 0.001
Figure 1E	Saline vs. D2 antagonist	Large 2 h	Response (%)	Sidak corrected Welch's *t*-test	*t*_(12)_ = –0.66, *P* = 0.522
Figure 1E	Saline vs. D2 antagonist	Small 2 h	Response (%)	Sidak corrected Welch's *t*-test	*t*_(12)_ = –0.5, *P* = 0.626
Figures 2F,G	All injection types	Count	Pauses in cue responding	One-way ANOVA	*F*_(6, 6)_ = 5.49, *P* ≤ 0.001
Figures 2F,G	All injection types	Cumulative time (s)	Pauses in cue responding	One-way ANOVA	*F*_(6, 37)_ = 10, *P* ≤ 0.001
Figure 2F	Saline vs. D1 agonist low dose	Count	Pauses in cue responding	Sidak corrected Welch's *t*-test	*t*_(12)_ = –0.159, *P* = 0.876
Figure 2F	Saline vs. D1 agonist high dose	Count	Pauses in cue responding	Sidak corrected Welch's *t*-test	*t*_(12)_ = –3.941, *P* = 0.012
Figure 2F	Saline vs. D1 antagonist	Count	Pauses in cue responding	Sidak corrected Welch's *t*-test	*t*_(12)_ = 3.158, *P* = 0.04
**Figure**	Drug (s)	Variables	Event	Test	Result
Figure 2F	Saline vs. D1 agonist low dose	Cumulative time (s)	Pauses in cue responding	Sidak corrected Welch's *t*-test	*t*_(12)_ = 0.94, *P* = 0.616
Figure 2F	Saline vs. D1 agonist high dose	Cumulative time (s)	Pauses in cue responding	Sidak corrected Welch's *t*-test	*t*_(12)_ = 4.498, *P* = 0.006
Figure 2F	Saline vs. D1 antagonist	Cumulative time (s)	Pauses in cue responding	Sidak corrected Welch's *t*-test	*t*_(12)_ = –3.89, *P* = 0.006
Figure 2G	Saline vs. D2 agonist low dose	Count	Pauses in cue responding	Sidak corrected Welch's *t*-test	*t*_(11)_ = –0.805, *P* = 0.876
Figure 2G	Saline vs. D2 agonist high dose	Count	Pauses in cue responding	Sidak corrected Welch's *t*-test	*t*_(11)_ = –1.317, *P* = 0.642
Figure 2G	Saline vs. D2 antagonist	Count	Pauses in cue responding	Sidak corrected Welch's *t*-test	*t*_(12)_ = 1.917, *P* = 0.316
Figure 2G	Saline vs. D2 agonist low dose	Cumulative time (s)	Pauses in cue responding	Sidak corrected Welch's *t*-test	*t*_(11)_ = 1.069, *P* = 0.616
Figure 2G	Saline vs. D2 agonist high dose	Cumulative time (s)	Pauses in cue responding	Sidak corrected Welch's *t*-test	*t*_(11)_ = 4.618, *P* = 0.006
Figure 2G	Saline vs. D2 antagonist	Cumulative time (s)	Pauses in cue responding	Sidak corrected Welch's *t*-test	*t*_(12)_ = –4.541, *P* = 0.006
Figures 4E,J	Saline	1 vs. 2 h	Component 1-Component 2	Paired Wilcoxon signed-rank test	*P* < 0.05
Figure 4E	D1 agonist low dose	1 vs. 2 h	Component 1-Component 2	Paired Wilcoxon signed-rank test	*P* > 0.1
Figure 4E	D1 agonist high dose	1 vs. 2 h	Component 1-Component 2	Paired Wilcoxon signed-rank test	*P* > 0.1
Figure 4E	D2 agonist high dose	1 vs. 2 h	Component 1-Component 2	Paired Wilcoxon signed-rank test	*P* > 0.1
Figure 4E	D2 agonist low dose	1 vs. 2 h	Component 1-Component 2	Paired Wilcoxon signed-rank test	*P* > 0.1
Figure 4E	D2 agonist high dose	1 vs. 2 h	Component 1-Component 2	Paired Wilcoxon signed-rank test	*P* > 0.1
Figure 4E	Saline vs. D1 agonist low dose	1 h	Component 1-Component 2	Non-Paired Wilcoxon signed-rank test	*P* > 0.1
Figure 4E	Saline vs. D1 agonist low dose	2 h	Component 1-Component 2	Non-Paired Wilcoxon signed-rank test	*P* > 0.1
Figure 4E	Saline vs. D1 agonist high dose	1 h	Component 1-Component 2	Non-Paired Wilcoxon signed-rank test	*P* < 0.05
Figure 4E	Saline vs. D1 agonist high dose	2 h	Component 1-Component 2	Non-Paired Wilcoxon signed-rank test	*P* < 0.05
Figure 4E	Saline vs. D2 agonist low dose	1 h	Component 1-Component 2	Non-Paired Wilcoxon signed-rank test	*P* > 0.1
Figure 4E	Saline vs. D2 agonist low dose	2 h	Component 1-Component 2	Non-Paired Wilcoxon signed-rank test	*P* < 0.1
Figure 4E	Saline vs. D2 agonist high dose	1 h	Component 1-Component 2	Non-Paired Wilcoxon signed-rank test	*P* < 0.05
Figure 4E	Saline vs. D2 agonist high dose	2 h	Component 1-Component 2	Non-Paired Wilcoxon signed-rank test	*P* < 0.05
Figure 4J	D1 antagonist	1 vs. 2 h	Component 1-Component 2	Paired Wilcoxon signed-rank test	*P* > 0.1
Figure 4J	D2 antagonist	1 vs. 2 h	Component 1-Component 2	Paired Wilcoxon signed-rank test	*P* > 0.1
Figure 4J	Saline vs. D1 antagonist	1 h	Component 1-Component 2	Non-Paired Wilcoxon signed-rank test	*P* < 0.05
Figure 4J	Saline vs. D1 antagonist	2 h	Component 1-Component 2	Non-Paired Wilcoxon signed-rank test	*P* > 0.1
Figure 4J	Saline vs. D2 antagonist	1 h	Component 1-Component 2	Non-Paired Wilcoxon signed-rank test	*P* < 0.05
Figure 4J	Saline vs. D2 antagonist	2 h	Component 1-Component 2	Non-Paired Wilcoxon signed-rank test	*P* > 0.1
Figure 5A	All injection types	Onset latency (s)	Cued Movement	One-way ANOVA	*F*_(4, 37)_ = 1.06, *P* = 0.395
Figure 5A	All injection types	Latency (s)	Receptacle entry	One-way ANOVA	*F*_(4, 27)_ = 0.43, *P* = 0.784
Figure 5B	All injection types	Efficiency	Cued Movement	One-way ANOVA	F4,27 = 1.19, *P* = 0.339
Figure 5C	saline	Rate in 1 and 2 h	Receptacle entry	Repeated Measures ANOVA	*F*_(1, 6)_ = 3.46, *P* = 0.112
Figure 5C	D1 agonist low dose	Rate in 1 and 2 h	Receptacle entry	Repeated Measures ANOVA	*F*_(1, 6)_ = 4.76, *P* = 0.072
Figure 5C	D1 agonist high dose	Rate in 1 and 2 h	Receptacle entry	Repeated Measures ANOVA	*F*_(1, 6)_ = 4.94, *P* = 0.068
Figure 5C	D2 agonist low dose	Rate in 1 and 2 h	Receptacle entry	Repeated Measures ANOVA	*F*_(1, 5)_ = 5.05, *P* = 0.075
Figure 5C	D2 agonist high dose	Rate in 1 and 2 h	Receptacle entry	Repeated Measures ANOVA	*F*_(1, 5)_ = 2.43, *P* = 0.18
Figure 5C	Saline vs. D1 agonist low dose	Rate (Hz) pre-cue	Receptacle entry	Sidak corrected Welch's *t*-test	*t*_(12)_ = 0.068, *P* = 1
Figure 5C	Saline vs. D1 agonist low dose	Rate (Hz) post-cue	Receptacle entry	Sidak corrected Welch's *t*-test	*t*_(12)_ = –0.635, *P* = 1
Figure 5C	Saline vs. D1 agonist high dose	Rate (Hz) pre-cue	Receptacle entry	Sidak corrected Welch's *t*-test	*t*_(12)_ = –0.875, *P* = 1
Figure 5C	Saline vs. D1 agonist high dose	Rate (Hz) post-cue	Receptacle entry	Sidak corrected Welch's *t*-test	*t*_(12)_ = –0.681, *P* = 1
Figure 5C	Saline vs. D2 agonist low dose	Rate (Hz) pre-cue	Receptacle entry	Sidak corrected Welch's *t*-test	*t*_(11)_ = 0.494, *P* = 1
Figure 5C	Saline vs. D2 agonist low dose	Rate (Hz) post-cue	Receptacle entry	Sidak corrected Welch's *t*-test	*t*_(11)_ = –0.46, *P* = 1
Figure 5C	Saline vs. D2 agonist high dose	Rate (Hz) pre-cue	Receptacle entry	Sidak corrected Welch's *t*-test	*t*_(11)_ = –1.039, *P* = 1
Figure 5C	Saline vs. D2 agonist high dose	Rate (Hz) post-cue	Receptacle entry	Sidak corrected Welch's *t*-test	*t*_(11)_ = –0.599, *P* = 1
Figure 5D	saline	ITI length (10 s bins)	Response (%)	Repeated measures ANOVA	*F*_(5, 5)_ = 2.04, *P* = 0.107
Figure 5D	D1 agonist high dose	ITI length (10 s bins)	Response (%)	Repeated measures ANOVA	*F*_(5, 5)_ = 0.71, *P* = 0.621
Figure 5D	D2 agonist high dose	ITI length (10 s bins)	Response (%)	Repeated measures ANOVA	*F*_(5, 5)_ = 1.24, *P* = 0.318
Figure 5E	Saline	Path length (cm)	Response type	Repeated measures ANOVA	*F*_(1, 6)_ = 21.13, *P* = 0.004
Figure 5E	D1 agonist low dose	Path length (cm)	Response type	Repeated measures ANOVA	*F*_(1, 6)_ = 13.51, *P* = 0.01
Figure 5E	D1 agonist high dose	Path length (cm)	Response type	Repeated measures ANOVA	*F*_(1, 6)_ = 1.91, *P* = 0.216
Figure 5E	D2 agonist low dose	Path length (cm)	Response type	Repeated measures ANOVA	*F*_(1, 5)_ = 51.96, *P* ≤ 0.001
Figure 5E	D2 agonist high dose	Path length (cm)	Response type	Repeated measures ANOVA	*F*_(1, 5)_ = 6.69, *P* = 0.049
Figure 5E	Saline vs. D1 agonist low dose	Path length (cm)	Trials with a response	Sidak corrected Welch's *t*-test	*t*_(12)_ = 0.995, *P* = 0.678
Figure 5E	Saline vs. D1 agonist low dose	Path length (cm)	Trials with no response	Sidak corrected Welch's *t*-test	*t*_(12)_ = –0.01, *P* = 1
Figure 5E	Saline vs. D1 agonist high dose	Path length (cm)	Trials with a response	Sidak corrected Welch's *t*-test	*t*_(12)_ = –5.123, *P* ≤ 0.001
Figure 5E	Saline vs. D1 agonist high dose	Path length (cm)	Trials with no response	Sidak corrected Welch's *t*-test	*t*_(12)_ = –4.743, *P* ≤ 0.001
Figure 5E	Saline vs. D2 agonist low dose	Path length (cm)	Trials with a response	Sidak corrected Welch's *t*-test	*t*_(11)_ = –0.826, *P* = 0.678
Figure 5E	Saline vs. D2 agonist low dose	Path length (cm)	Trials with no response	Sidak corrected Welch's *t*-test	*t*_(11)_ = –0.049, *P* = 1
Figure 5E	Saline vs. D2 agonist high dose	Path length (cm)	Trials with a response	Sidak corrected Welch's *t*-test	*t*_(11)_ = –2.558, *P* = 0.081

*Each row describes one statistical comparison using the data in the graph indicated by “Figure.” “Drugs” describes the drug condition(s) being compared (an entry of an individual drug means that the comparison is made only with data obtained after injection of that drug); “Variables” and “Event” together describe the measures being compared; “Test” names the statistical test used, and “Result” provides the results of the test*.

**Figure 2:**
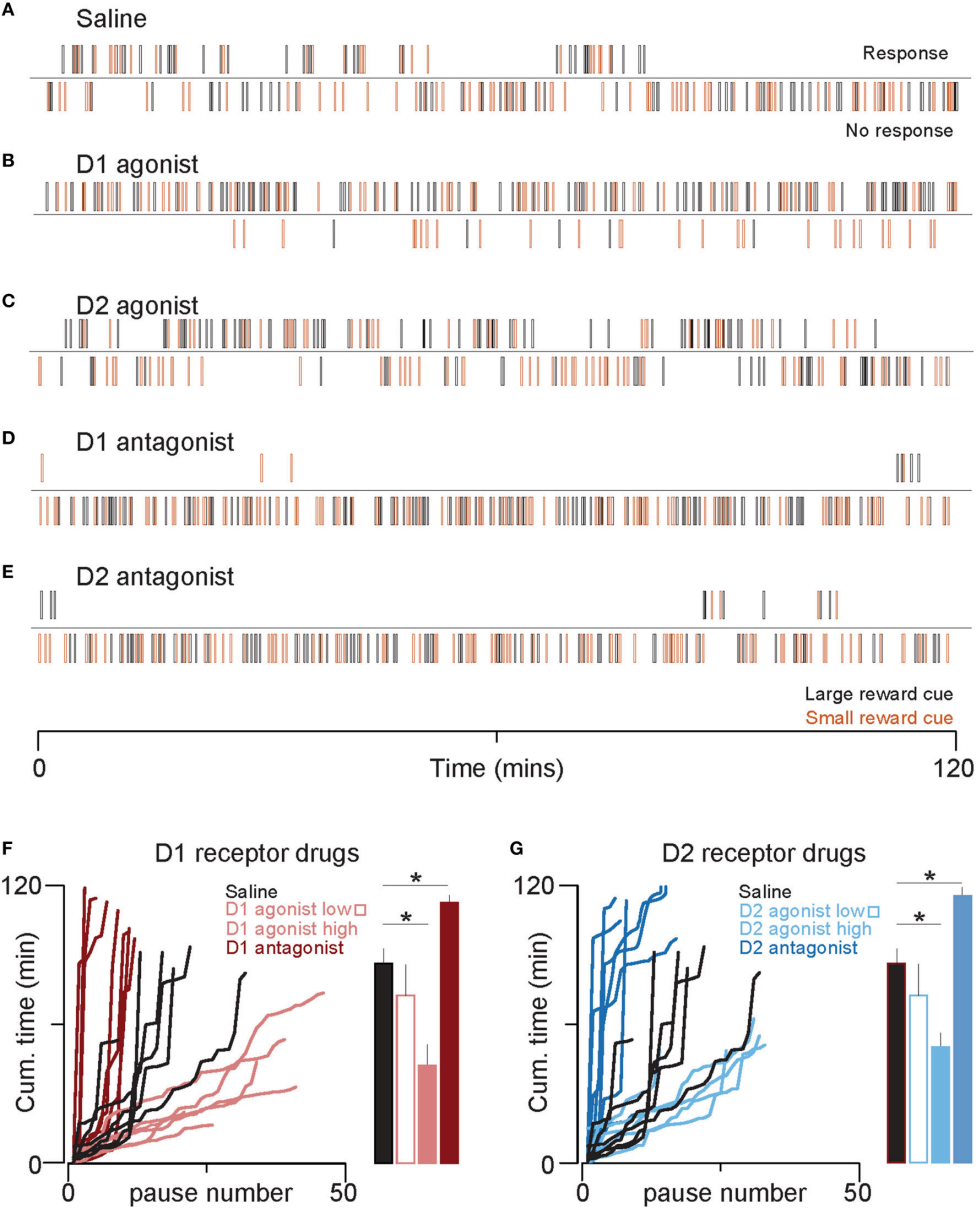
**D1 and D2 receptor agonists decrease time spent in the non-responsive state**. **(A–E)** Rasters show five example sessions, one for each drug (high doses only). Each line represents the time at which a cue predicting large (black) or small reward (orange) was presented. The top raster of each pair indicates cues that the animal responded to by entering the receptacle. The bottom raster indicates cues that the animal did not respond to. Note that the length of the non-responsive state is longer toward the end of the session in the control condition **(A)**, but the non-responsive state is very short or absent in the D1 agonist **(B)** and D2 agonist **(C). (F)** The left graph plots cumulative time spent in the non-responsive state against the number of transitions from responsive to non-responsive. Thus, steeper lines indicate long pauses (non-responses to contiguous sequences of cues) interrupted with few responses and shallower lines represent short pauses with frequent responses. Each line is the data from an individual rat. The bar plots on the right show the mean cumulative time spent in a non-responsive state over the entire session for each treatment group. Color conventions are identical to those in Figure 1D. **(G)** The graphs follow identical conventions to those in **(F)**, but here for the D2 agonist and antagonist treatments. Color conventions are the same as those in Figure 1E. ^*^p < 0.05.

In Figures 2F,G, cues with no response were first flagged, and “pauses” were defined as ≥2 successive trials with no response. The pause length was defined as the time interval between cues with responses. The cumulative time spent in pauses is plotted against the sequential pause number (left panels), and the mean cumulative time spent in pauses through the end of the session is shown in the bar plots (right panels). One-way ANOVAs with drug type as a factor were used to assess whether the number of pauses or the cumulative time spent in pauses differed between drugs. *Post-hoc* two-tailed Sidak-corrected Welch's *t*-tests were used to compare both pause number and total time spent in pauses in each drug and saline.

**Figure 3:**
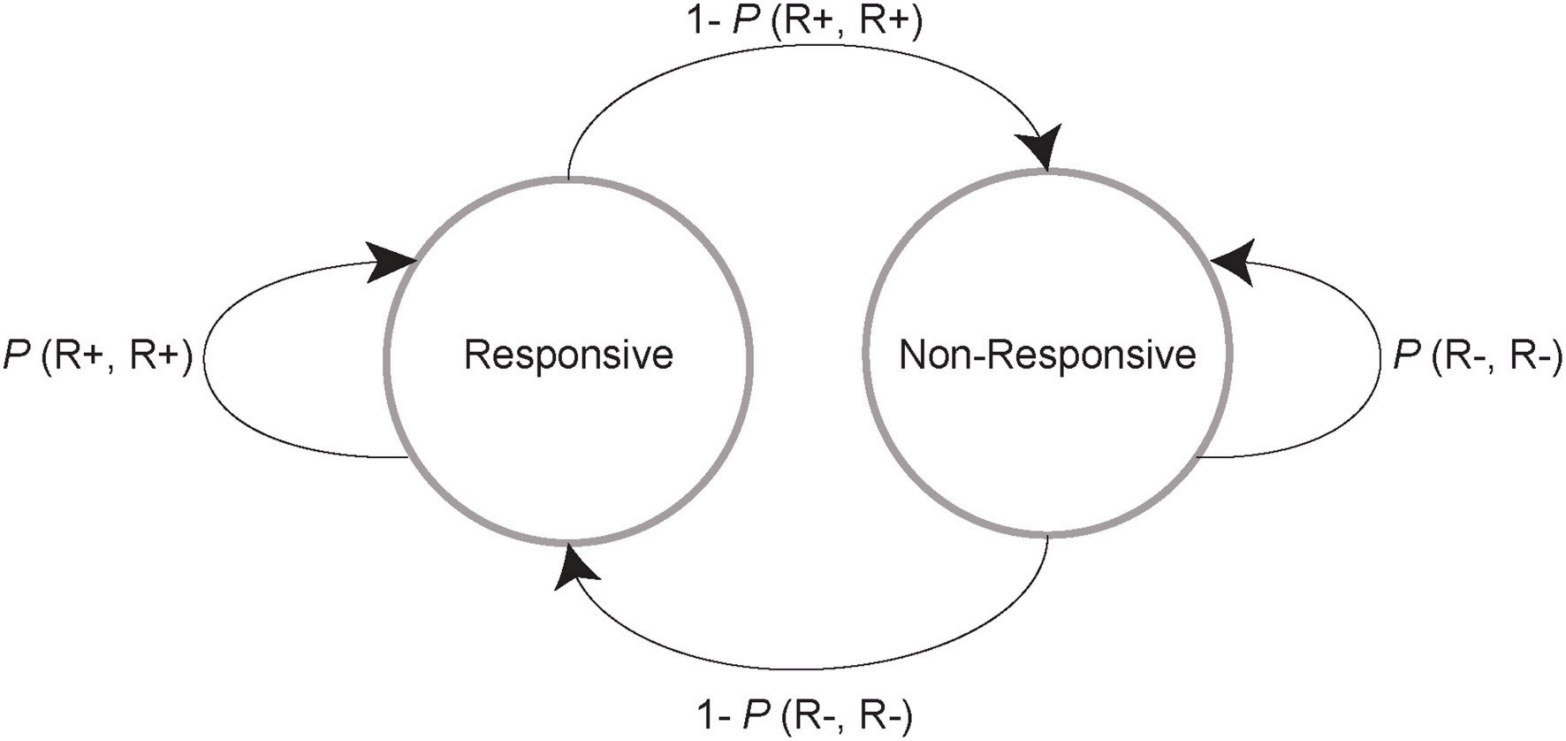
**Schematic of a two-state Markov model**. On a given trial, a rat can either stay in the responsive (left circle and looping arrow) or non-responsive state (right circle and looping arrow) or transition to the other state (arrows between the circles). Each of these events occurs with a probability that is calculated by classifying all trials as those in which the animal responded (R+) and did not respond (R−), and then classifying the next trial in the same way. This procedure results in 4 possible categories for any consecutive pair of trials: R+,R+; R+,R−; R−,R−; and R−,R+. The probability of the second trial outcome given the first is determined with the equation *P*_(*Response type* 1, *Response type* 2)_ = N_(*Response type* 1, *Response type* 2)_ / N_(*Response type* 1)_, where N is the number of trials. The 4 categories that describe the possible behaviors in any pair of trials form a stochastic matrix. We resolved this matrix into steady state probability vectors, which yield an estimate of the probability of finding each subject in either the responsive or non-responsive state at a steady state of the Markov chain.

**Figure 4:**
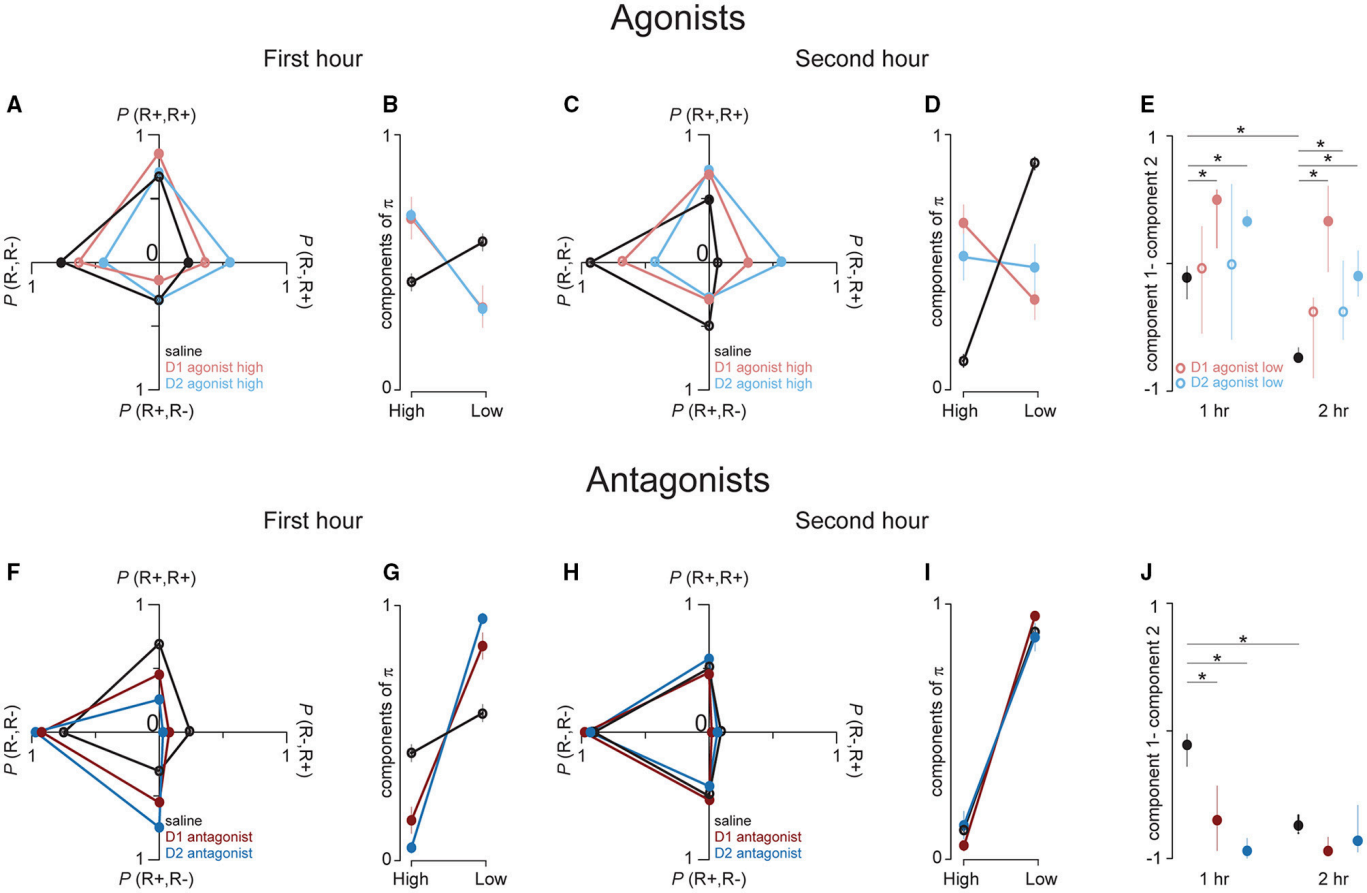
**D1 and D2 receptor agonists promote transistions from the non-responsive to responsive state. (A,C,F,H)**. These graphs show the associated transition probabilities for all 4 possible response/no response pairs, calculated with the equation given in the legend to Figure 3. **(A)** The dots represent the transition probabilities in the first hour of behavioral testing for saline (black), high dose D1 agonist (red) and high dose D2 agonist (blue) treated rats. Note that in this first hour there is a very high probability of responding to a cue if the rat responded to the previous cue; this is indicative of response clustering. **(B)** The dots represent the cross-session mean of the components of the probability vectors calculated from the transition matrices that compose the mean probabilities in **(A)**. The two components give a steady state estimate of the probability of the rats being in the high (“High,” left dots) and low (“Low” right dots) responsive state, respectively. D1 agonist data is almost completely obscured by the D2 agonist data. **(C,D)** This data comes from the same sessions as in **(A,B)**, but the data is taken from the second hour of testing. **(E)** Dot plots show the median and middle quartiles of the differences between the two components of the probability vectors that are shown in **(B)** (left set of dot plots) and **(D)** (right dot plots). Filled symbols represent saline and high agonist doses. Open symbols represent low agonist doses (corresponding data in **(A–D)** are not shown for low doses). **(F–J)** Same plotting conventions as in **(A–E)**, but these figures show data from the first **(F,G)** and second hour **(H,I)** after saline (black), D1 antagonist (dark red) and D2 antagonist (dark blue) injections. ^*^p < 0.05.

In **Figures 4A,C,F,H**, each trial *t* was coded as eliciting a response (R+) or failing to elicit a response (R−). We then calculated the empirical probability of the occurrence of R+ or R− at *t*+1. This procedure results in 4 probability measures, each of which is associated with a unique pattern of response and no response on the two consecutive trials, *t* and *t*+1: *P*_(R+R+)_, *P*_(R+R−)_, *P*_(R−R−)_, *P*_(R−R+)_. When these probabilities are arranged so that each couplet that begins with the same response type (R+ or R−) is on the same row of a 2 × 2 matrix, each row sums to one; i.e., the matrix is right stochastic. In **Figures 4A,C,F,H**, we plotted (separately for each drug) the mean probabilities for each couplet with the row values of these matrices on the same axis. For example, *P*_(R+R+)_, *P*_(R+R−)_ are on the vertical axis because each couplet begins with an R+. Because each row of each matrix sums to one, the matrix values are all positive, and the rat can freely transition from a responsive (R+) to non-responsive state (R−), and vice versa, the stochastic matrix can describe a Markov chain for which a stationary probability vector π can be calculated. These probability vectors are estimates of the probability of finding the rat in the responsive and non-responsive state at a steady state of the Markov chain (Figure 3). To calculate the components of π, we transposed each matrix, found the left eigenvalues of the transposed matrices and then divided these values by their sum (which ensures that the components of π sum to 1). The mean probability vector for each treatment group is plotted in Figures 4B,D,G,I. Thus, we have two unique ways of characterizing behavior: by the stochastic matrix, which graphically shows mean transition probabilities, and by the vector of stationary probabilities, which yields an estimate of the probability that the rat is in either the responsive or non-responsive state. To compare these probability vectors across drugs and time, we subtracted the two components of π, an approach that preserves information about the relative direction of the pair of probability estimates. In Figures 4E,J, we plotted the cross-session median and middle quartiles of these differences within each drug separately for each session hr. To determine for each drug whether these probability vectors differed between the first and second hour of the sessions, we compared their differences with paired Wilcoxon signed rank tests. Next, we performed non-paired Wilcoxon signed rank tests (saline vs. drug) within each hr and corrected the 6 p values (one for each drug vs. saline) with a Sidak correction.

**Figure 5:**
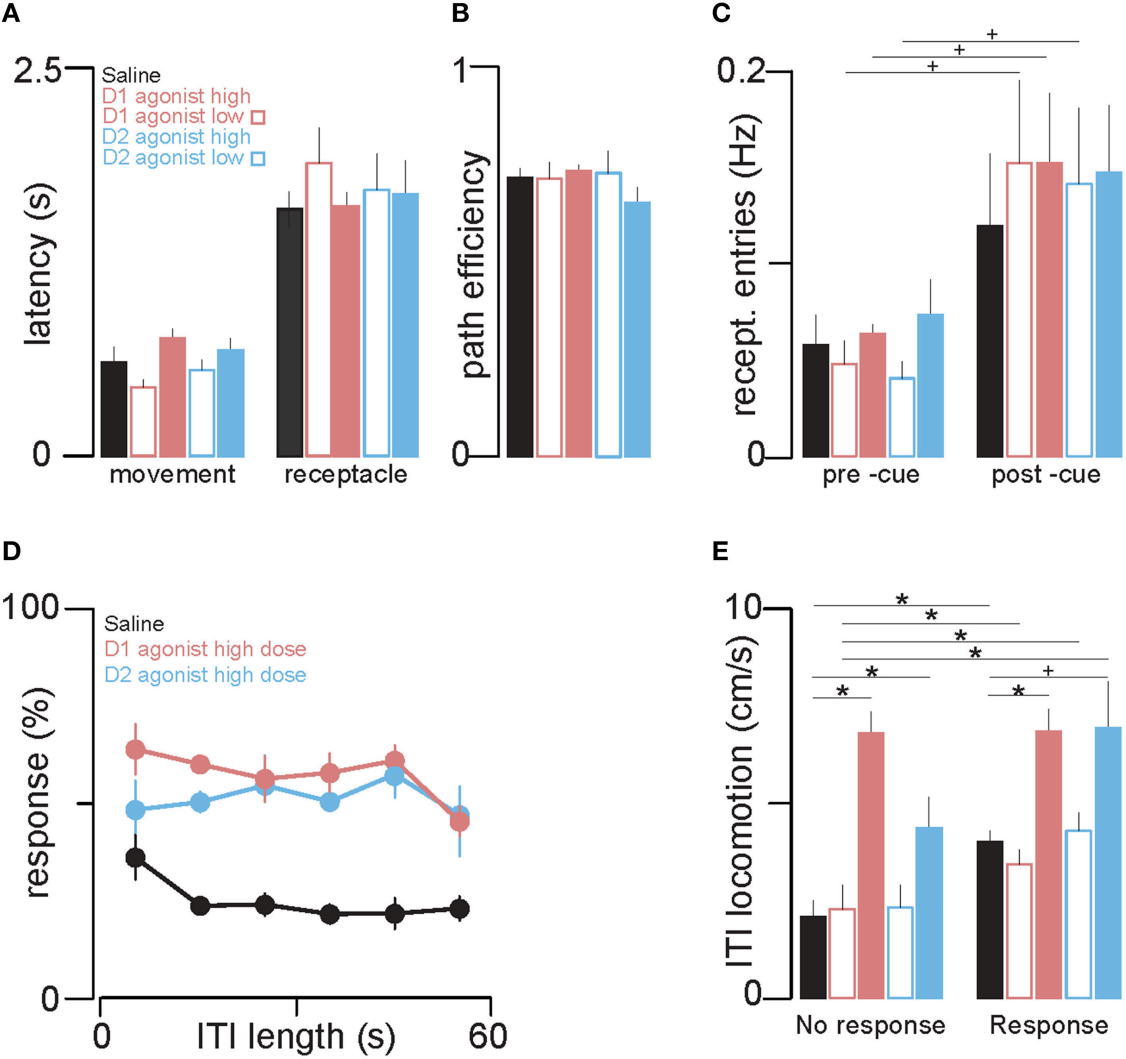
**The dopamine agonists increase locomotion, but increased cue responding is not attributable to increased locomotion. (A)** The left group of bars shows the effects of injection of saline, D1 and D2 agonists on the mean latency to initiate movement after cue onset, and the right group shows the mean latency to reach the receptacle. Color conventions are the same as in Figures 1D,E. **(B)** Path efficiency (a measure of directness of approach to the receptacle) was not affected by either agonist or saline. **(C)** The mean rate of receptacle entry 5 s before (left group of bars) and during cue presentation (right group) for trials with a behavioral response. **(D)** The mean response ratio (%) for different ITI lengths (bin width = 10 s) after saline (black), D1 agonist (light red) and D2 agonist (light blue) injection. The probability of a cued response was not significantly correlated with ITI length in any of the treatment groups. **(E)** The bars show the mean rate of locomotion during ITIs when rats respond (left group of bars) and do not respond (right group) to the subsequent cue. ^*^p < 0.05, ^+^*p* < 0.1.

In Figures 5A,B, cues to which the animal responded were first isolated. In Figure 5A, the latencies of the animal to begin movement directed toward the receptacle (left bars) and to reach the reward receptacle (right bars) were calculated and plotted as the cross-session mean. In Figure 5B, we calculated, for each trial, the length of the path (in cm) that the animal took to the receptacle from its position at cue onset. We then calculated the ratio of two values: (A) the straight-line distance between the rat's position at cue onset and the receptacle, and (B) the length of the actual path taken to reach the receptacle. These A:B ratios are termed “path efficiency” values; they range from 0 to 1, with values closer to 1 indicating more efficient (less circuitous) paths. Path efficiencies were plotted as cross-session means for each drug type. To assess whether each of these latency values or the path efficiency measure differed between drugs, we performed one way ANOVAs with drug as a factor. In Figure 5C, for each trial with a rewarded receptacle entry we counted the number of receptacle entries 5 s prior to cue onset and 5 s after cue onset. These counts were then converted to rates (entries per s) by summing them over all rewarded trials in the session and dividing this value by the number of rewarded trials multiplied by 5 s (the longest possible trial length). The cross-session mean rates for each drug are shown in the bar plots in Figure 5C. To compare these two rates, for each drug, we used repeated measures ANOVA with time interval (pre and post cue intervals) as an independent variable. To compare receptacle entry rates between saline and drug within each time interval, we performed Sidak-corrected Welch's *t*-tests. In Figure 5D, we sorted trials by the preceding inter-trial interval (ITI) length and grouped these values into 10 s bins. We then calculated response ratios for trials with ITIs that fell within each bin and calculated the cross-session mean for each drug. We used ITI bin number as a factor in a repeated measures ANOVA to assess whether, in each drug, response probability varied across ITI durations. In Figure 5E, for each trial we calculated the total distance traveled (in cm) during the ITI preceding cue onset. Then we calculated the within-session mean distance traveled in the ITIs preceding cues to which the animal responded, and similarly for cues to which the animal did not respond. To assess whether total distance traveled differed between trials with and without a subsequent cued response, within each drug we used repeated measures ANOVA with response type as a factor. Next, we performed *post-hoc* Sidak-corrected Welch's *t*-tests to compare average path lengths traveled for each response type (drug vs. saline).

### Histology

**Figure 6:**
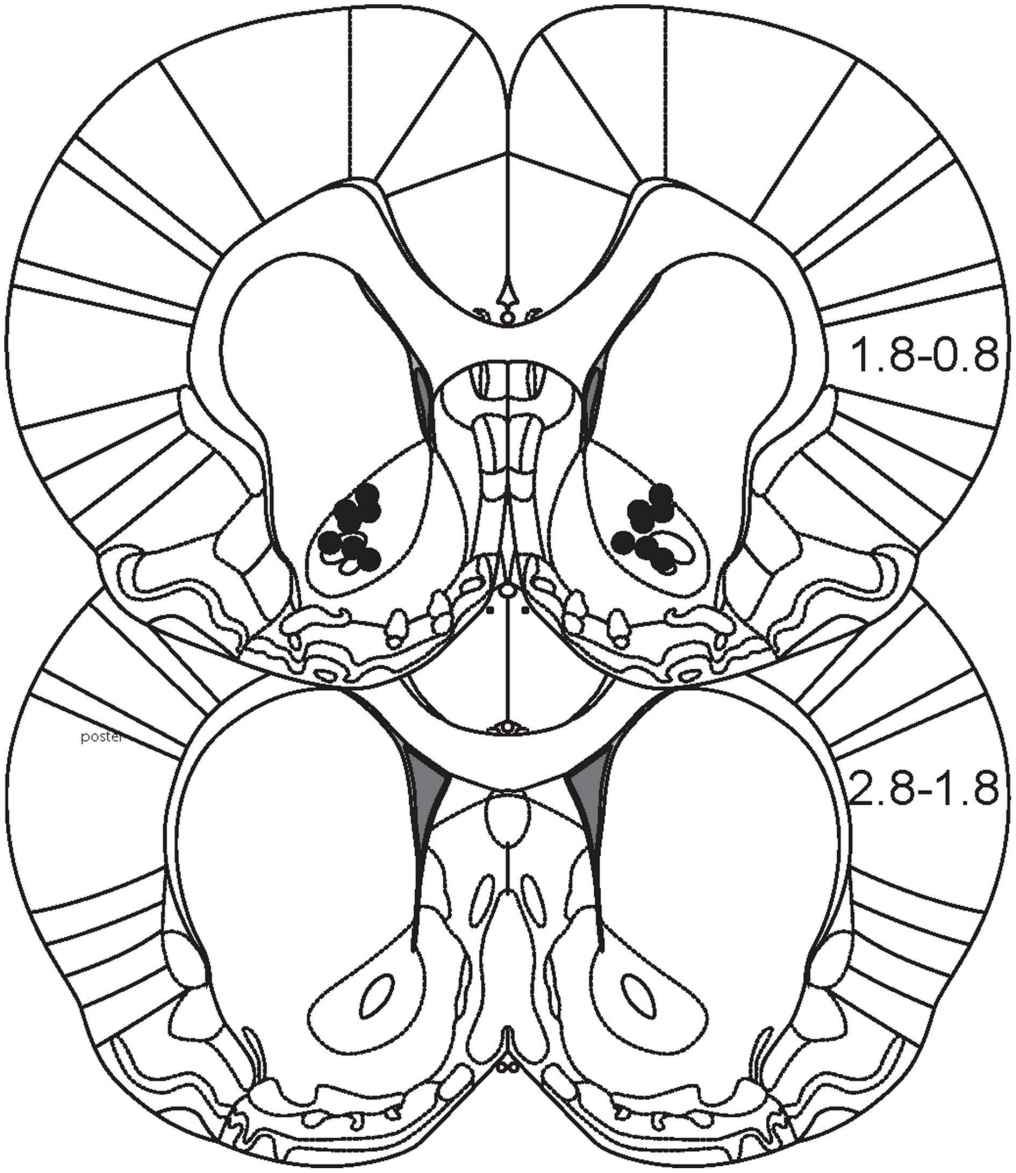
**Histological reconstruction of injection sites**. Figure depicts two coronal sections of rat brain that encompass the majority of the anterior-posterior extent of the NAc (0.8–2.8 mm anterior from Bregma). Black dots represent estimates of the location of microinjections for each animal.

Animals were deeply anesthetized with Euthasol and decapitated with a guillotine. Brains were quickly removed from the skull and then fixed in formalin. Prior to slicing with a cryostat, brains were cryoprotected by immersion in 30% sucrose for several days. Sections (50 μm) were stained for Nissl substance to visualize cannula and injector tracks. Estimates of injection sites for each animal are shown in Figure 6.

## Results

### Response probability

We trained 8 rats to respond to distinct auditory cues that predicted either a small or large sucrose reward (Figure 1A). Even though the animals were not food-restricted, they responded to nearly every cue predictive of 10% liquid sucrose (Figures 1B,C, black lines) while not substantially discriminating between large (Figure 1B) and small (Figure 1C) reward availability. In contrast, from the first day that sucrose reward concentration was reduced from 10% to 3%, a pronounced run-down of cued responses was observed across the 2 h of testing (Figures 1B,C, gray lines). There are at least two possible explanations for this effect. First, it could be due to a state of satiety as animals accrue nutrient with successive cue responses. However, this is unlikely because nutrient accrues faster with 10% than 3% sucrose rewards of the same volume, yet the run-down was far more pronounced with 3% sucrose. The second possibility, which we favor, is that whereas 10% sucrose is sufficiently reinforcing to maintain responding throughout the session, equivalent volumes of 3% sucrose are not. Whatever its cause, the run-down effect allowed us to ask whether activation of dopamine receptors using exogenous agonists increases the response ratio. This question cannot be answered using 10% sucrose rewards or in food-restricted animals because baseline responding is close to 100% in those conditions and thus cannot be increased.

By the time performance stabilized, 4 days after switching to 3% sucrose rewards, a difference in responding to large and small reward cues was evident toward the beginning of the session (compare Figure 1B with Figure 1C); this difference dwindled as the session progressed and responding to both cue types declined. This significant difference between large and small cue responding is also evident in the average response ratio over the first hour of the session after saline (vehicle control) injections in the NAc: subjects responded to 54 ± 5% of large reward-associated cues and to 33 ± 3% of small reward-associated cues (Figures 1D,E, left black circles). The probability of responding to both cues was lower in the second hour; moreover, the response ratio for the large and small cues was statistically indistinguishable during this period (Figures 1D,E, right black circles; see Table 1 for statistical results). Therefore, animals responded more to cues that predict large than small rewards only in the first half of the session.

To examine the temporal pattern of responding in greater detail, we constructed raster plots that show the time of each cue presentation and whether the animal responded (top raster, Figure 2A) or not (bottom raster). As shown in an example session prior to which saline had been injected, both responses and failures to respond typically occurred in clusters of several successive cues (Figure 2A). This suggests that there are two states that dictate response probability: responsive and non-responsive. Furthermore, as the session progressed, the reduction in response probability was due to longer periods of time spent in the non-responsive state (Figure 2A, top raster). To quantify the changing duration of the non-responsive states, we plotted, for each session, the cumulative time spent in the paused (non-responsive) state against the sequential pause number. In essentially all saline injection sessions, these lines became steeper toward the end of the session, indicating that individual non-responsive states became longer as the sessions progressed (Figures 2F,G, black lines).

To assess the contribution of NAc core dopamine to the decision to respond to reward-predictive cues, we pharmacologically increased or decreased D1 or D2 dopamine receptor signaling by microinjecting the D1 receptor agonist SKF 81297 or antagonist SCH 23390, or the D2 receptor agonist quinpirole or antagonist raclopride. We found that both D1 and D2 agonists significantly increased responding to cues (Figure 1D, light red squares; Figure 1E, light blue squares); in particular, the low dose of each agonist increased responding only in the second hour, whereas the high doses increased responding across the entire session (Figure 1D, light open red squares; Figure 1E, light open blue squares). Generally, responding to large and small reward cues was increased to roughly equivalent degrees, and this was the case for both D1 and D2 receptor agonists (Figures 1D,E and Table 1).

These increases in response ratio were accompanied by a different pattern of responding compared with saline-treated animals (Figures 2B,C). In contrast to the control condition, where the time spent in the non-responsive state increased as the session progressed, the responses of agonist-treated animals was comparatively sustained for the entire session, with brief but relatively frequent transitions to the non-responsive state (Figure 2F, D1 agonist, light red lines; Figure 2G, D2 agonist, light blue lines). Both agonists significantly reduced the cumulative time spent in the non-responsive pause state and largely prevented the steep increase in the cumulative time spent in pauses that occurred in the second hour of the session in saline-treated animals.

Antagonists to both D1 and D2 receptors had the opposite effect of the agonists. The antagonists strongly reduced responding to cues in the first half of the session, while leaving responding in the second half unchanged (likely due to a floor effect) (Figure 1D), dark red triangles; (Figure 1E, dark blue triangles). Both antagonists also significantly prolonged the cumulative time spent in the non-responsive state (Figures 2D,E,F,G).

### Transition probabilities

The increase in cue responses caused by the D1 and D2 agonists, as well as the greater time spent in the responsive than the non-responsive state, could be explained either by an increased probability of transitioning from the non-responsive to the responsive state, or conversely, a decreased probability of transitioning from the responsive to the non-responsive state (or both). To determine which of these was the case we implemented a simple two state Markov model (Figure 3) by calculating empirical transition probability matrices for the 4 possible pairs of successive events: two successive cued responses (R+R+), a response to a cue followed by a non-response to the next cue (R+R−), a non-response followed by a response (R−R+), and a non-response followed by a non-response (R−R−). Note that R+R+ and R−R−correspond to remaining in the responsive and non-responsive states, respectively; and that R+R− and R−R+ correspond to transitioning from one state to another. The probability of each of these pairs of outcomes was computed by dividing the number of times the pair occurred in a given time window (e.g., the first hour of the session) by the number of times the first member of the pair occurred (e.g., *P*_(R+R−)_ = N_(R+R−)_ / N_(R+)_; see Methods section Data analysis). Note that the probability of transitioning out of a state is therefore 1 minus the probability of remaining in a state (e.g., *P*_(R+R−)_ = 1 – *P*_(R+R+)_). Thus, in Figures 4A,C,F,H, the data on the vertical axis of the left graphs show the average (across rats) probability of maintaining or transitioning out of the responsive state, whereas the data on the horizontal axis shows the probability of maintaining or transitioning out of the non-responsive state.

In the first hour of behavioral testing, saline-treated rats tended to cluster their cue responding: if they responded to one cue, the probability of a response to the next cue was greater than that of a non-response (*P*_(R+R+)_ > *P*_(R+R−)_; Figure 4A, vertical axis); conversely, if they did not respond to a cue, the probability of a non-response to the next cue was greater than that of a response (*P*_(R−R−)_ > *P*_(R−R+)_; Figure 4A, horizontal axis). Treatment with either the D1 or D2 agonist did not strongly change the probability of remaining in the responsive state (R+R+) [or, equivalently, the probability of transitioning to the non-responsive state (R+R−)] compared to saline injections (Figure 4A, vertical axis). However, agonist-treated animals transitioned significantly more frequently from the non-responsive to the responsive state (and, equivalently, remained in the non-responsive state less frequently; Figure 4A, horizontal axis).

In the second hour of the session, saline-treated rats showed a marked decrease in the probability that they would transition from the non-responsive to the responsive state compared to the first hour (Figure 4C vs. Figure 4A, horizontal axis). Moreover, they were more likely to transition from the responsive to the non-responsive state in the second hour than the first (Figure 4C vs. Figure 4A, vertical axis). Therefore, as the session progressed, under control conditions the decline in responding (Figures 1B,D) was due to both longer non-responsive states and shorter responsive states. Treatment with either D1 or D2 agonists shifted the response probabilities in the second hour along both axes (Figure 4C). Therefore, whereas in the first hour the agonists increased the likelihood of transitioning out of the non-responsive state without affecting transitions out of the responsive state, in the second hour, the agonists both increased transitions out of the non-responsive state and decreased transitions out of the responsive state—meaning that the agonists both increased the length of responsive states and decreased the length of non-responsive states. Notably, these effects of the agonists caused the second-hour transition probabilities to resemble those in the first hour in the control condition. That is, the agonists prevented the decline in responding in the second hour by preventing the normal shift toward transition probabilities that favored the non-responsive state.

Both the D1 and the D2 antagonist shifted responding in the first hour along both axes, indicating that they encouraged transitions toward the non-responsive state as well as prevented transitions to the responsive state (Figure 4F). Intriguingly, in the second hour, the transition probabilities in antagonist and in saline were nearly identical (Figure 4H), and the transition probabilities in antagonist-treated animals were not significantly different in the first and second hour (Figure 4F vs. Figure 4H). These results indicate that D1 and D2 antagonists induce, in the first hour, a set of transition probabilities that is nearly identical to that which normally occurs in the second half of the session in control conditions, corresponding to long stretches of non-responsiveness to cues.

To statistically compare these transition probabilities in drug and saline, we resolved each matrix into probability vectors; i.e., we estimated, from the transition matrices, the probability of each rat in each condition being in a responsive and non-responsive state at the steady state of a Markov chain (see Methods, section Data analysis, and Figure 3). In Figures 4B,D, it is evident that in the control (saline) condition, the probability distributions for the responsive and non-responsive state strongly shift toward the non-responsive state in the second hour. In contrast, these probabilities are relatively stable in both agonists across the entire session. In antagonist (Figures 4G,I), the distribution of the probabilities of each state are strongly shifted toward the non-responsive state in both hr and these probabilities are nearly identical to those in the second hour in saline treated animals. In Figures 4E,J we subtracted, for each session hr and each drug, the components of the probability vectors shown in Figures 4B,D,G,I. Thus, values above and below zero indicate a greater probability of being in the responsive and non-responsive state, respectively. During the first hour in saline, there was a near equal probability of being in the responsive and non-responsive states. In the second hour, this distribution of state probabilities significantly shifted toward the non-responsive state (Figure 4E, left black dots vs. right black dots). In the high dose of either agonist, there was a significant increase in the probability of being in the responsive state in the first hour compared to saline (Figure 4E, left dots) and this was maintained in the second hour of the session (Figure 4E, right dots). Thus, constitutive activation of dopamine receptors is sufficient to promote and maintain the responsive state under conditions of normative satiety. The antagonists had the opposite effect; they strongly and significantly shifted the state probability distributions toward the non-responsive state in both the first and second session hour. Furthermore, there was no difference statistically between the state probability distributions in antagonist and in saline during the second hour of the session. Thus, blocking dopamine receptor activation induces a non-responsive state with the same efficacy as task experience over time in the control condition. Furthermore, activation of these same receptors powerfully promotes a transition to the responsive state to cues that predict food reward even in the absence of caloric need.

### Cued and uncued locomotion

It is possible that the agonist effects resulted from greater non-directed receptacle entries due to a non-specific increase in locomotion rather than an increase in receptacle-directed approach responses. To compare these hypotheses, we used video tracking data to examine post-cue movement parameters on trials where the animal responded to the cue. There was no statistically significant difference between control and agonist treated sessions in the latency to initiate locomotion after cue onset (Figure 5A, left bars) or the latency to reach the receptacle (Figure 5A, right bars). In addition, the path efficiency of the cued movement (the ratio of the length of a straight line between the animal and the receptacle to the length of the path the animal actually followed) was not changed by agonist treatments (Figure 5B). Because non-directed, random movements resulting in receptacle entry would be expected to be less direct (and therefore less efficient) and/or to occur at longer latency, these observations suggest that the agonist-treated animals made directed movements toward the reward receptacle after cue onset in a manner similar to their cued approach movements in saline.

We next assessed whether agonist-induced increases in cued entries could have been due to a non-specific increase. Examining only trials with a response, we compared the rate of receptacle entries in the 5 s prior to cue onset to the rate of entry in the 5 s after cue onset. The agonists did not significantly increase the average rate of either spontaneous or cued entries (Figure 5C) which suggests that receptacle entry remains under cue control in agonist. Together, the results in Figures 5A–C demonstrate that the increase in probability of cued approach caused by the agonists is not attributable to non-specific factors such as an increase in non-directed locomotion or the rate of uncued receptacle entries.

### Locomotion during ITIs

Although the agonist-induced increase in cued responding was not attributable to an increase in non-directed locomotion, this conclusion does not preclude the possibility that the agonists nevertheless induced a concomitant increase in locomotion not directed toward the receptacle. To quantify locomotion during the ITI, we first asked whether the probability of a cue response varied as a function of ITI length. As shown in Figure 5D, response ratio (collapsed across large and small cues) was fairly constant across the entire range of ITI lengths in both agonist and saline. Next, we calculated average distance traveled per s of the ITI for each of the treatment groups, and compared this rate of locomotion across trials where rats responded and did not respond to the subsequent cue. Intriguingly, in the control (saline) condition, there was significantly more locomotion during ITIs followed by a cued receptacle approach (Figure 5E, right black bar) than when the animals failed to make a subsequent cued receptacle approach (Figure 5E, left black bar). These results suggest that uncued locomotion occurs with greater frequency when the animal is in the responsive state.

To determine whether this process involves dopamine receptor activation in the NAc, we assessed the effects of the dopamine agonists on locomotion during the ITI. The D1 agonist significantly increased locomotion during ITIs both with and without a subsequent response; similarly, the D2 agonist caused either a significant increase (no-response trials) or a trend to an increase (response trials) (Figure 5E). Thus, the dopamine agonists caused an overall increase in locomotion during the ITIs. In the presence of agonists, this locomotion occurred at similarly high levels whether or not the animal subsequently responded, suggesting that ITI locomotion is more sensitive to dopamine receptor activation than cue responding. In sum, the results shown in Figure 5 suggest that, via a mechanism within the NAc, dopamine receptor activation biases animals both toward higher probability of responding to cues and higher rates of spontaneous locomotion, and that even though dopamine has both of these effects, the higher response probability driven by dopamine is not a spurious consequence of higher rates of spontaneous locomotion.

## Discussion

### NAc dopamine is necessary and sufficient for cued taxic approach

Cue-elicited approach is strongly dependent on the mesolimbic dopamine projection from the VTA to the NAc only in very specific circumstances: those in which responding involves “flexible approach” (Nicola, 2010) [also called “taxic” (Petrosini et al., 1996) or “guidance” (O'keefe and Nadel, 1978) approach; the term “taxic approach” will be used here]. Taxic approach refers to locomotion that is directed toward a visible object from starting locations that vary across approach occasions. Importantly, taxic approach requires the brain to compute a novel movement path for each approach event [unlike “praxic,” “orientation,” or “inflexible” approach, which occurs when the starting and ending locations are constant across approach events (O'keefe and Nadel, 1978; Petrosini et al., 1996; Nicola, 2010)]. The present study extends the conclusion that NAc dopamine is required for taxic approach in four ways. First, whereas the dependence of taxic approach on mesolimbic dopamine was first established using a discriminative stimulus (DS) task that required the animal to approach an operandum (lever or nose poke) to obtain sucrose reward delivered into a nearby receptacle (Yun et al., 2004a,b; Ambroggi et al., 2008; Nicola, 2010), in the present task, animals had simply to approach the reward receptacle itself. As in the DS task, cues were presented at long and variable intervals, resulting in diverse starting locations at cue onset due to the animal's movement about the chamber during the intertrial interval (not shown)—conditions under which approach behavior is necessarily taxic. Our observation that D1 and D2 dopamine receptor antagonist injection into the NAc core reduced the proportion of cues to which the animal responded parallels earlier observations with the DS task (Yun et al., 2004a,b; Ambroggi et al., 2008; Nicola, 2010). Similar to earlier findings with a progressive delay task (Wakabayashi et al., 2004), our results confirm, in a much simpler task, that inclusion of an explicit operant contingency at a location that differs from the reward delivery site is not a critical task feature that renders taxic approach behavior dependent on NAc dopamine.

Second, whereas earlier studies were conducted in food-restricted animals, the present work demonstrates that taxic approach is impaired by NAc dopamine antagonist injection even in animals given *ad libitum* access to chow. The dependence of taxic approach on mesolimbic dopamine is therefore not a function of nutrient deficit or the subject's state of hunger. Indeed, the present results support a role for mesolimbic dopamine in promoting cue-elicited approach to high-calorie food even in the absence of a homeostatic need for calories, supporting the hypothesis that this circuitry contributes to overeating and obesity (Berridge et al., 2010; Kenny, 2011; Stice et al., 2013; Meye and Adan, 2014).

Third, whereas previous studies used dopamine antagonists to show that NAc dopamine is necessary for cued taxic approach, in the present work we demonstrate that increasing NAc D1 or D2 dopamine receptor activation by injection of agonists of these receptors is sufficient to increase the probability that a cue will elicit taxic approach. This experiment was not possible in most previous studies because food-restricted rats respond to close to 100% of cues that reliably predict nutrient, imposing a ceiling on potential agonist effects. However, when sucrose prediction was made less reliable in a “probabilistic stimulus” (PS) task in which the PS predicted 10% sucrose reward on only 15% of trials, the response probability was lower, and pharmacological blockade of dopamine reuptake increased this probability (Nicola et al., 2005). In the present study, rats were fed chow *ad libitum* and the reward for cue responding was 3% instead of 10% sucrose. Under these conditions, even though the cues reliably predicted reward, animals responded to a smaller fraction of cues than under food-restricted or 10% sucrose conditions, eliminating the ceiling effect and allowing us to assess the effects of agonists on cued taxic approach. Consistent with the results from the PS task, dopamine agonist injection in the NAc core produced a robust increase in cue responding. The present results therefore establish that NAc core dopamine receptor activation is both necessary and sufficient to promote cued taxic approach, supporting our previous conclusion that mesolimbic dopamine is part of the causal mechanism for taxic approach initiation (du Hoffmann and Nicola, 2014).

Fourth, our observation that D1 and D2 agonists have very similar effects that are the opposite of the effects of D1 and D2 antagonists has important implications for conclusions about the specificity of the drugs' effects. In most previous studies, microinjected D1 and D2 antagonists had very similar behavioral (Hiroi and White, 1991; Ozer et al., 1997; Koch et al., 2000; Yun et al., 2004b; Eiler et al., 2006; Pezze et al., 2007; Lex and Hauber, 2008; Liao, 2008; Nicola, 2010; Shin et al., 2010; Haghparast et al., 2012; Steinberg et al., 2014) and electrophysiological (du Hoffmann and Nicola, 2014) effects. Because the concentration of injected antagonists required to observe effects is much higher than the binding constants of these drugs for their target receptors, the similarity of D1 and D2 antagonist effects calls into question their specificity: it is possible that the drugs either bind to the same dopamine receptor, or to a third receptor class that is not a dopamine receptor at all. In the former case, activating one of the receptors should produce no behavioral effect; in the latter case, activating neither receptor should produce a behavioral effect. However, we find that D1 and D2 agonists both produce behavioral effects, and that their effects are identical to each other and precisely opposite to those of the antagonists. It would be remarkable if 4 different drugs all acted at the same off-target receptor. Therefore, the more likely scenario is that all of the drugs act specifically at their target receptors.

### Effects of dopamine agonists are not due to a generalized increase in locomotion

A potential complication with the interpretation that the dopamine agonists promoted cue responding is that the effect could have been due to a generalized increase in locomotion, resulting in spurious receptacle entries that would have occurred whether or not a cue had been presented. Indeed, in the control condition, video tracking data obtained during the session revealed that locomotion rate during the intertial interval was correlated on a trial-by-trial basis with receptacle entry probability during the subsequent cue presentation. Furthermore, the agonists increased both locomotion during the inter-trial intervals and cue response probability. One way to rule out a generalized motor effect is to use a non-reward predictive stimulus (NS) to show that responding to NS presentation is not increased by the agonists. We did not include an NS in our design. We hypothesize that had we done so, we would have observed an increase in locomotion during the NS (as occurred during the intertrial interval) but not an increase in receptacle entries. This hypothesis is based on several observations indicating that the increased entry probability after cue presentation was not a result of increased generalized locomotion. First, the increase in locomotion during the inter-trial interval caused by the agonists was decoupled from the increase in cue responding, occurring even during intervals that were followed by a non-response to the cue (Figure 5E). Second, the probability of an uncued receptacle entry during the ITI was not increased by the agonists (Figure 5C). Finally, compared with directed entries, entries resulting from a generalized increase in locomotion would be expected to occur at longer latency after cue onset, and the animal would be expected to follow a more circuitous path from its location at cue onset to the receptacle; however, the agonists neither increased cue-entry latencies (Figure 5A) nor decreased movement path efficiency (Figure 5B). Together, these results indicate that the increase in cued receptacle entries caused by the agonists is not due to the concomitant increase in locomotion. A more likely explanation is that some spontaneous locomotor events were taxic approaches toward objects within the chamber, and the probability of such approaches was increased by the agonists just as the probability of taxic approach in response to our explicitly-presented cues was increased.

### Lack of a pronounced difference in responding to cues predicting large and small reward

Another difference between the current task and our previous studies using DS and PS tasks is that we presented two reward-predictive cues, which predicted large and small volumes of sucrose, rather than one reward-predictive cue and one non-reward-predictive stimulus (NS). We included dual reward-predictive cues in the task design in order to assess whether manipulations of NAc dopamine receptors differentially influence behavior triggered by cues predictive of different reward magnitudes. However, we could not conduct such an analysis because the animals did not robustly differentiate between the two cues. When the reward was 10% sucrose, there were no significant differences in response ratio between large- and small-reward predictive cues; and when the reward was 3% sucrose, a small (~20%) difference was observed only in the first hour of the session (Figure 1). These observations contrast with typical behavior in the DS task using exactly the same auditory stimuli, in which animals respond to >80% of DS presentations and < 10% of NS presentations (Nicola, 2010). More recently, we found that in a task similar to the present one, using the same two auditory stimuli but with one cue predictive of reward contingent on receptacle entry and one NS, responding to the NS was quite high (>20%; not shown). This high responding (compared to the low NS response ratio in DS tasks with an explicit operant requirement) is likely due to some degree of generalization between the predictive and non-predictive cues, as well as to the lack of an operant response contingency. The absence of such a contingency means that cue responding is less difficult and requires less effort than cue responding in the DS task, potentially explaining the difference in NS response probability. If >20% response ratios for a NS are common, then they should be even higher when the cue predicts a small amount of reward, precisely as observed in the present study.

### Decline in responding over time may be an extinction-like effect

A striking feature of the behavior observed in our *ad libitum* chow-fed animals was a decline in cue response probability over the 2 h session, which was far more pronounced when the reward was 3% sucrose than when it was 10% sucrose. Rats given free access to sucrose show a similar decline in lick rate from the beginning of the session, which is attributable to satiation: post-ingestive nutrient detection mechanisms signal to the brain, resulting in reduced consumption (Smith, 2001). However, satiation is unlikely to account for the decline in cue responding observed here because the greater nutrient intake when 10% sucrose was the reward would be expected to produce a more rapid decline in responding than when 3% sucrose was delivered, yet the opposite occurred (Figure 1). Another possible explanation is that the decline is an extinction-like effect that is due to delivery of reinforcers that are of insufficient magnitude to maintain responding to the cues on subsequent trials. Although we have no direct evidence that this is the case, simply ceasing to deliver sucrose also results in a decline in responding (not shown). Although this true extinction effect is more rapid than that observed here, the slower time course of extinction in the present case would be expected because a small amount of sucrose was delivered. Moreover, when a higher concentration of sucrose (10%) was delivered, almost no decline was observed, consistent with the idea that 3% sucrose reinforcers were of insufficient magnitude to maintain responding.

That 3% sucrose is less reinforcing than 10% is hardly surprising, given not only that 3% sucrose is less preferred over water than 10% (Sclafani, 1987), but also that 10% sucrose is likely to more strongly activate post-ingestive processes that detect nutrient intake, which can contribute to reinforcement even in the absence of taste (de Araujo et al., 2012; Sclafani and Ackroff, 2012; Sclafani, 2013; de Araujo, 2016). These processes promote dopamine signaling and in fact appear to be responsible for the ability of nutritive sucrose reinforcers to sustain progressive ratio task performance to a far greater extent than sweet non-nutritive reinforcers (Beeler et al., 2012). Indeed, cues predictive of sucrose elicit more dopamine release in the NAc than cues predictive of non-nutritive sweetner (McCutcheon et al., 2012) and, under some conditions, sucrose itself elicits more dopamine release than sweetner (Beeler et al., 2012). These results suggest that an attenuated dopamine signal during 3% sucrose sessions (vs. 10%) could be responsible for the extinction-like decline in responding when the lower sucrose concentration was used.

Consistent with this hypothesis, activation and inhibition of dopamine receptors interacted with the extinction-like effect. D1 or D2 dopamine receptor agonist injection both increased the initial (first hour) rate of responding and greatly reduced the magnitude of the normal decline in responding from the first to second hour compared with the control condition (Figures 1D,E), essentially preventing the extinction-like effect. In contrast, D1 or D2 antagonist injection reduced the response rate in the first hour of the session to values indistinguishable from those normally observed in the second hour, essentially mimicking and/or accelerating extinction. One possibility is that NAc core dopamine is part of the reinforcement mechanism that prevents extinction. This idea is consistent with the proposed role for dopamine as a reward prediction error signal, which is thought to be the basis for learned changes in the neural representation of value predicted by stimuli (Montague et al., 1996; Schultz et al., 1997; Schultz, 1998). It is also consistent with a role for dopamine in “reboosting” such value representations (Berridge, 2012). On the other hand, dopamine agonists would be expected to constitutively activate dopamine receptors, thereby mimicking so-called “tonic” dopamine; although the agonists would activate dopamine receptors at the time that reward is delivered, they would also activate the receptors to a similar degree at all other times. It is difficult to conceptualize how such a constant signal could be interpreted as a prediction error or as a “reboosting” signal that serves to indicate that a discrete reinforcing event has occurred.

An alternative hypothesis is that the dopamine drugs did not interfere with reinforcement, but with a neural mechanism that directly activates cued approach behavior. This proposal is supported by our previous studies demonstrating that a large proportion (nearly half) of NAc neurons are excited by cues in a DS task (Ambroggi et al., 2008; McGinty et al., 2013; du Hoffmann and Nicola, 2014; Morrison and Nicola, 2014); furthermore, in a cued receptacle approach task similar to the one used here (i.e., without an operant response contingency), a similar proportion of NAc neurons is excited (Caref and Nicola, 2014). Using video tracking, we established that these excitations precede the onset of approach locomotion and predict the latency at which it will occur (McGinty et al., 2013; du Hoffmann and Nicola, 2014; Morrison and Nicola, 2014). Moreover, injection of dopamine antagonists into the NAc reduced the magnitude of these excitations while impairing the ability to initiate cued approach (du Hoffmann and Nicola, 2014). These results suggest that dopamine directly facilitates the cue-evoked excitations of NAc neurons that drive approach, perhaps by rendering them more excitable in response to glutamatergic input (Nicola et al., 2000, 2004; Hopf et al., 2003). Thus, treatment of NAc neurons with dopamine receptor agonists may have increased the probability of cued approach behavior by mimicking an excitatory neuromodulatory effect of endogenous dopamine and thereby increasing the magnitude of cue-evoked excitations.

### Clustered response pattern may be due to fluctuations in tonic dopamine levels

Another feature of the animals' task performance is that responses and non-responses to cues were not randomly distributed, but appeared to be clustered into bursts of several consecutive responses or non-responses. In the control (vehicle injection or no injection) conditions, response clusters were longer and more frequent toward the beginning of the session, becoming shorter and less frequent toward session end; and necessarily vice-versa for non-response clusters. This pattern suggest that there are two states, responsive and non-responsive (Figure 3), which fluctuate with a time course of minutes, and which shift from an initial bias toward the responsive state to a later bias toward the non-responsive state. Dopamine agonist injection promoted the responsive state by decreasing the probability of transitioning to the non-responsive state (lengthening response clusters) and increasing the probability of transitioning to the responsive state (shortening non-response clusters), whereas antagonists had the opposite effect. The most striking consequences of the agonist effects occurred in the second hour of the session, when the drugs appear to have prevented the normal increased bias toward the non-responsive state: the second hour transition probabilities continued to resemble those in the first hour rather than shifting toward favoring the non-responsive state. In contrast, the antagonists had their greatest effects in the first hour, when they caused the transition probabilities to favor the non-responsive state, similar to the transition probabilities normally occurring in the second hour.

The effects of the dopamine agonists and antagonists on transition probabilities are consistent with the hypothesis that response state is a function of dopamine receptor occupation. Thus, when NAc dopamine levels reach and exceed a threshold, the animal is in the responsive state; below this threshold, the animal is in the non-responsive state. Testing this hypothesis would require measuring tonic dopamine levels as animals perform this or a similar task; the hypothesis predicts that dopamine levels should be higher during response clusters than non-response clusters. Although to our knowledge previous microdialysis studies have not examined whether fluctuations in dopamine level correlate with local taxic approach probability, a previous investigation found that NAc dopamine levels were higher when food pellets were dropped into receptacles at 45 s or 4 min intervals (both conditions likely necessitating taxic approach to obtain the food on each trial) than when food was freely available (a situation that minimizes the need for taxic approach) (McCullough and Salamone, 1992). Studies that have varied operant response rate requirements have produced somewhat conflicting results, with some observing a positive correlation between rate of operant responding and dopamine level (McCullough et al., 1993; Sokolowski et al., 1998; Cousins et al., 1999) and others finding exceptions to this proposed relationship (Salamone et al., 1994; Cousins and Salamone, 1996; Ahn and Phillips, 2007; Ostlund et al., 2011). A potential explanation for this discordance is that different operant tasks engage the need for taxic approach to different degrees (Nicola, 2010); correlations with dopamine level may be more robust for taxic approach probability than for operant response rate.

A related proposal is that tonic dopamine levels not only drive faster rates of responding (or perhaps greater probability of taxic approach), but also that dopamine levels are set by the rate of reinforcement (Niv et al., 2005, 2007), an idea that has recently gained experimental support (Hamid et al., 2016). Accordingly, dopamine levels in animals working for nutritive reinforcers should be lower in *ad libitum*-fed than in hungry animals [as is in fact the case (Ostlund et al., 2011)], and lower when the reinforcer is 3% sucrose than when it is an equivalent volume of 10% sucrose. The proposed low dopamine levels in 3% sucrose could result in a chain reaction, with low dopamine resulting in a low probability of responding to any given cue; failures to respond in turn drives the reinforcement rate and hence dopamine level still lower, and hence response probability on the next cue presentation also becomes lower. The result would be a progressive reduction in response rate similar to that observed here.

### Conclusions: Cued taxic approach is a model for investigation of regulation of mesolimbic dopamine by nutrient state

The low dopamine-dependent response probability in *ad libitum*-fed animals observed here is consistent with many recent studies of regulation of dopamine neurons by messengers, such as cholecystokinin, orexin, ghrelin, leptin, insulin and glucagon-like peptide 1, that signal the body's nutrient status detected via various mechanisms. In general, signals that report nutrient deficit increase dopamine neuronal activity, whereas signals that report satiety or nutrient surfeit decrease it (Ladurelle et al., 1997; Helm et al., 2003; Krügel et al., 2003; Abizaid et al., 2006; Fulton et al., 2006; Hommel et al., 2006; Narita et al., 2006; Kawahara et al., 2009; Leinninger et al., 2009; Quarta et al., 2009, 2011; Jerlhag et al., 2010; Perry et al., 2010; Domingos et al., 2011; España et al., 2011; Skibicka et al., 2011, 2012a,b, 2013; Davis et al., 2011a,b; Mebel et al., 2012; Patyal et al., 2012; Egecioglu et al., 2013; Cone et al., 2014, 2015; Mietlicki-Baase et al., 2014). The exquisite sensitivity of mesolimbic dopamine signaling to nutrient state is consistent with the proposal that the probability of mesolimbic dopamine-dependent behavior can change instantly as a result of the value, relative to the nutrient state, of the reinforcer (Berridge, 2012). We observe that low value reinforcers delivered to relatively sated animals result in fluctuating response probabilities superimposed on an overall decline in response probability. These observations, coupled with the dramatic shifts in response and transition probabilities produced by injection of dopamine agonists and antagonists into the NAc, suggest that, under our conditions, the dopamine level is held at low levels by nutrient sensing mechanisms. The control of dopamine levels by these and other parameters (such as recent reinforcement rate) may produce dopamine levels that fluctuate around the threshold for eliciting a response, causing cue responses and non-responses to occur in clusters. The behavioral paradigm we use here—mesolimbic dopamine-dependent sucrose-reinforced cued taxic approach in *ad libitum*-fed animals—is therefore ideal for further investigation of the regulation of dopamine dynamics by nutrient state, reinforcement rate, and other parameters, and of the mechanism by which these variables impact NAc dopamine-dependent behavior.

## Author contributions

JD designed and conducted the experiment, analyzed the data, and co-wrote the paper. SN advised JD on design and analysis and co-wrote the paper.

### Conflict of interest statement

The authors declare that the research was conducted in the absence of any commercial or financial relationships that could be construed as a potential conflict of interest.
